# Python-based geometry preparation and simulation visualization toolkits for STEPS

**DOI:** 10.3389/fninf.2014.00037

**Published:** 2014-04-11

**Authors:** Weiliang Chen, Erik De Schutter

**Affiliations:** Computational Neuroscience Unit, Okinawa Institute of Science and TechnologyOkinawa, Japan

**Keywords:** STEPS, stochastic reaction diffusion simulation, Python, geometry preparation, simulation visualization, tetrahedral mesh, Stochastic Simulation Algorithm

## Abstract

STEPS is a stochastic reaction-diffusion simulation engine that implements a spatial extension of Gillespie's Stochastic Simulation Algorithm (SSA) in complex tetrahedral geometries. An extensive Python-based interface is provided to STEPS so that it can interact with the large number of scientific packages in Python. However, a gap existed between the interfaces of these packages and the STEPS user interface, where supporting toolkits could reduce the amount of scripting required for research projects. This paper introduces two new supporting toolkits that support geometry preparation and visualization for STEPS simulations.

## Introduction

Advanced research on neuronal signaling pathways frequently requires assistance from computational modeling and simulations, causing the development of several molecular reaction-diffusion simulators in recent years. In this domain, the general assumption of mass action kinetics in a well-mixed volume is often invalid, whilst stochasticity and spatiality have been demonstrated to play essential roles in regulating behaviors of the system (Santamaria et al., 2006; Antunes and De Schutter, 2012; Anwar et al., 2013). Several spatial stochastic reaction-diffusion simulators have been developed, following two fundamentally different approaches; particle-based and voxel-based. Particle-based simulators track the Brownian motion of individual molecules during the simulation, simulating reactions based on molecule collisions. MCell (Stiles and Bartol, 2001) and Smoldyn (Andrews, 2012) are two examples of such simulators. Voxel-based simulators partition the simulated geometry as a volume mesh formed by small cubes or tetrahedrons, called voxels or subvolumes, within which the laws of chemical kinetics determine changes of the number of molecules. Diffusion is then simulated as the transport of molecules from one subvolume to another. A commonly used approach in stochastic voxel-based simulators is Gillespie's Stochastic Simulation Algorithm (Gillespie, 1977), which can easily be extended to deal with diffusion, referred to as “spatial SSA” or “spatial Gillespie.” Simulators that fall into this category include MesoRD (Hattne et al., 2005) and NeuroRD (Kotaleski and Blackwell, 2010), which implement variations of SSA in cubic meshes.

STEPS, short for STochastic Engine for Pathway Simulation, is a GPL-licensed, reaction-diffusion simulator that implements a spatial extension of Gillespie's SSA in tetrahedral geometries (Hepburn et al., 2012). While mainly focusing on spatial stochastic signal pathway simulations, STEPS is also able to simulate stochastic/deterministic well-mixed models as well as 3D deterministic models in tetrahedral meshes.

One feature that distinguishes STEPS from other spatial SSA simulators is its extensive Python interface. Python (http://www.python.org/) is a dynamic programming language with many packages that are beneficial for scientific research, such as NumPy (http://www.numpy.org/) and SciPy (http://www.scipy.org/) for scientific computing, and Matplotlib (http://matplotlib.org/) for data plotting. The simplicity, readability and ultimate flexibility of the language have raised interest from the computational neuroscience community, where many simulators now support Python as their optional or even default user interfaces, including NEURON (Carnevale and Hines, 2006; Hines et al., 2009), NEST (Diesmann and Gewaltig, 2002; Eppler et al., 2008), MOOSE (Ray and Bhalla, 2008), and more. Efforts have also been devoted to the integration of these simulators through Python, such as PyNN (Davison et al., 2008), which aims to provide a Python-based description for neuronal network models that can be executed in several supported simulators without modification. It is well known that one disadvantage of using pure Python coding is the sacrifice of computing speed for flexibility. Pure Python modules are normally one to two orders of magnitude slower than their C/C++ equivalents due to its runtime interpretation. A general solution for this issue is to implement the computational intensive modules in C/C++ and expose its APIs to Python using SWIG (Beazley, 1996) or the Boost library (Karlsson, 2005). With this solution, efficiency is maintained as most of the computation is executed by compiled C/C++ code, yet users can still appreciate benefits granted from the flexible Python-based interface. STEPS used this approach in its development (Wils and De Schutter, 2009). Internally it is implemented in C/C++ for computational efficiency, while many of its APIs are exposed to Python using SWIG, including those for model description, simulation control and data access. The Python interface approach is significantly different from the non-interactive approach employed in other stochastic reaction-diffusion simulators where a formatted file, with full model description as well as simulation parameter settings, is used as the input of the simulation, and results are exported to an output file.

As a stochastic pathway simulation engine, the user interface of STEPS is mostly generic and focuses on simulation control and data access. Thus, STEPS users rely on the massive number of scientific Python packages to achieve varying research objectives, from simple plots of molecule distribution, to complicated results analysis. However, some customized toolkits are required to reduce the amount of scripting by the user. The collection of these Python-based, customized toolkits is called the “STEPS supporting environment,” part of which has been described previously (Hepburn et al., 2012).

This paper describes two new supporting toolkits in this environment: the geometry preparation toolkit handles production of geometry data for STEPS simulations, while the visualization toolkit provides runtime visualization of simulations. In the following sections we will describe the details of both toolkits, including their functionality and the underling mechanisms and development principles. We will also provide examples to showcase their use, and discuss plans for the future improvements of the toolkits and the overall supporting environment.

## Materials and methods

### Geometry preparation toolkit for STEPS

Geometry preparation is an important prerequisite for reaction-diffusion simulation. It involves multiple procedures, starting with “geometry construction,” where a surface/volume mesh, or a set of geometry boundary representations, is created. Substructures and specific regions in the geometry that are of research interest or require extra simulation controls are then identified in a procedure of “component identification.” This is followed by “model association,” where biochemical models are assigned to the geometry components. Finally, the outcomes of these procedures are integrated together and prepared for simulation.

Reaction-diffusion simulators commonly accept formatted text files as data input, where geometry is described either as a combination of predefined primitives like spheres and cubes, or as a surface or volume mesh. The data is then dealt with differently among simulators. SSA based simulators like MesoRD and NeuroRD generate cubic meshes according to the input primitive geometries, while particle based simulators like Smoldyn and MCell establish mathematical boundary representations of the geometries. Data files for simplified geometries can be produced manually, but the generation of complex or realistic geometries, like those based on reconstructions from electron microscopic imaging, often relies on third party professional applications. Therefore, toolkits that integrate the geometry generator and the simulator can be beneficial. One example is CellBlender (http://www.mcell.psc.edu/), a toolkit that integrates MCell with Blender (http://www.blender.org/), providing a complete solution for triangular surface mesh construction, component identification, MCell model association and simulation result visualization.

Different from MesoRD and NeuroRD, STEPS does not generate meshes itself, but makes use of professional mesh generators. A generic mesh importing mechanism is provided, together with importing functions for common mesh formats such as Abaqus (http://www.3ds.com/products-services/simulia/portfolio/abaqus/), TetGen (http://tetgen.berlios.de/), and Gmsh (http://geuz.org/gmsh/). To further enhance this interaction, we developed a Python-based toolkit that integrates STEPS with CUBIT (https://cubit.sandia.gov/), a sophisticated surface/volume mesh generator. CUBIT provides both commercial and academic licensing as well as a 30-day full trial version. There are several reasons that we choose CUBIT as the primary supporting application. Unlike MCell, which accepts triangular surface meshes as its geometry inputs and is thus able to utilize free surface mesh generators such as Blender, STEPS simulations require tetrahedral meshes that are not supported by those generators. Open source tetrahedral mesh generators such as TetGen and Gmsh remain focused on a non-interactive scripting based generation approach and are therefore unqualified for the mesh preparation tasks described here. CUBIT not only implements multiple tetrahedron mesh generation algorithms, from simple automatic approaches to complex, geometry adapting methods, but also embeds an interactive Python environment and a large set of Python base APIs, which enables flexible data and function integration with STEPS. It supports importing of multiple mesh formats including the Abaqus format, the primary mesh format used in STEPS. Additionally, CUBIT supports both primitive-based mesh generation that is suitable for simplified geometry generation, and a facet-based engine for realistic geometry reconstruction, and is therefore suitable for a wider range of research compared to other generators that support a single approach.

As mesh generation is mostly controlled by CUBIT itself, the geometry preparation toolkit focuses on facilitating the remaining procedures that support five major functionalities in CUBIT and STEPS.

#### Element selection in CUBIT

In STEPS, geometry components are identified as groups of tetrahedrons and triangles. Technically this means that to create a component one has to select mesh elements and produce a list of their indices. There are several ways to select mesh elements in CUBIT depending on the condition. For simple geometries, components can be predefined before mesh generation and used to guide the generation process. They can also be separated and identified easily by simple mathematical spacing after mesh construction. However, these approaches become inadequate as the complexity and irregularity of the geometry increase, where extra support is necessary to ensure accuracy and efficiency.

**Figure 1:**
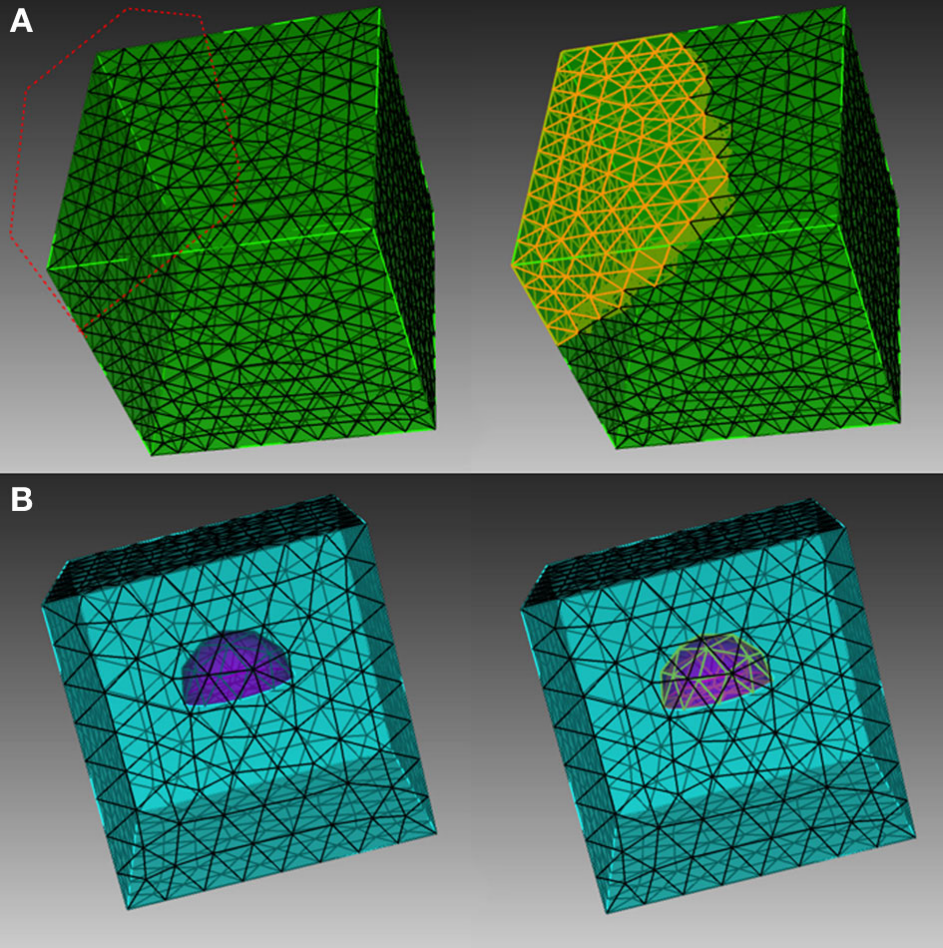
**Different element selection methods in CUBIT. (A)** Direct range selection of elements. Elements within the red dot line range are selected. **(B)** Indirect bounding object selection. Elements within the purple hemisphere are selected.

In order to explain the element selection mechanism, we first classify mesh elements into two different categories, skin and inner elements. Skin elements are directly visible from the outside, while inner elements are covered by skin elements and are thus not directly visible. In CUBIT, skin elements can be selected directly using box/polygon range selection. CUBIT also provides an x-ray option which, together with range selection, is able to select all elements within the range, regardless of whether they are skin or inner, as shown in Figure 1A. This method, however, cannot be used to select pure inner elements, because the covered skin elements are also selected. Our toolkit addresses this issue by implementing an indirect element selection method, which makes use of CUBIT's Python-based API. With this method, a bounding object is firstly created using CUBIT to virtually bound all the desired elements in the mesh. The toolkit then loops over all existing elements and opts for those that overlap with the bounding object by coordinate matching (Figure 1B). While mainly developed for inner element selection, this method can also be used to select elements within any arbitrarily created boundaries in general. In practice, the element selection approaches often have to be combined for different conditions.

#### Elementproxy object and index mapping in STEPS

Elements selected in CUBIT can be output as a Python list that contains their indices. However, for reasons of computational efficiency STEPS uses an internal indexing system, which is different from the one generated in CUBIT. Thus, a mapping mechanism is necessary for data enquiry and index conversion between the two systems. This mapping is handled by “ElementProxy,” a Python-based utility object implemented in STEPS. In short, the ElementProxy is a generic object for the storage of both geometry data as well as the mapping of indexing systems for a given type of geometry elements such as vertex, triangle and tetrahedron. During mesh import, coordinates, connectivity and other geometry data of an element are recorded in the associated ElementProxy object, together with its original index. The ElementProxy then assigns a STEPS index for the element and stores the mapping between them. The object is implemented purely in Python so that it can be archived and retrieved by the standard Pickle module in Python. This mechanism allows the toolkit to translate element indices between STEPS and CUBIT, which is necessary for the construction of the steps.geom.Tetmesh geometry object used for reaction-diffusion simulation in STEPS (Hepburn et al., 2012) and for other mesh preparation functionalities such as element highlighting.

#### Tetmesh geometry and region of interest datasets in STEPS

STEPS spatial geometry consists of two basic components: “compartment” and “patch.” A compartment, described by a collection of tetrahedrons in the mesh, is a 3D volume within which molecules can diffuse and react. A patch, described by a collection of triangles, is a 2D surface connected to one or two compartments, where molecules may be embedded. “Surface reaction” and “surface diffusion” can be assigned to a patch to describe membrane-related phenomena such as molecular channeling, transportation, and lateral diffusion. Compartments and patches can be based on lists of, respectively, tetrahedral and triangle indices from the element selection process shown in Figure 1, and then index mapping can be used to construct corresponding geometry components in the steps.geom.Tetmesh geometry.

Beside compartments and patches, smaller geometry element groups, for example, tetrahedrons that form a spine in a spiny dendrite, often need to be accessed repeatedly, either for the change of simulation parameters or for recording of results. In general, they can be stored as Python lists in an external file and loaded from the file during simulation. However, the manual creation and maintenance of external files can be exhausting, particularly for large simulations. Alternatively, the Tetmesh object provides an auxiliary “Region of Interest” (ROI) dataset where element lists can be named and stored. ROI datasets are accessible by name once created. A set of ROI operation APIs are also implemented in STEPS so that stored elements can be reused in the simulation.

#### Biochemical model association in STEPS

To form a spatial reaction-diffusion system, groups of reaction and diffusion rules (“volume systems”) defined in the biochemical model need to be added to corresponding compartments in the geometry, and groups of defined surface reaction rules and other surface phenomena (“surface systems”) need to be added to related patches. Volume systems and surface systems are defined separately in a steps.model.Model object. STEPS associates biochemical systems with geometry components by storing system ids in corresponding components in the geometry object. The model and geometry objects are then combined to construct the stochastic spatial solver (steps.solver.Tetexact). The separation of biochemical model definition and geometry description not only helps modelers to maintain focus, but also enhances the reusability of scripts as a single model definition can be reused with different geometries, and vice versa.

#### Mesh input and output in STEPS

In practice, biochemical model and geometry are often prepared by different individuals, therefore it is necessary for a Tetmesh to be stored in a file and retrieved later for simulation. This functionality is provided by the MeshIO utility, which saves and loads a Tetmesh object, compartment and patch definitions, biochemical model association and lists of element groups, to and from an xml file.

Though geometry preparation can be accomplished manually using the above mechanisms, the toolkit combines these mechanisms and provides flexible pipeline functions in Python that significantly reduce the labor required. For example, selected tetrahedrons in CUBIT can be directly used to create a compartment with biochemical system association in Tetmesh geometry within a single function call in the toolkit, instead of going through the steps of index translation, compartment object creation and model association. This is particularly beneficial when using complex geometries.

### Visualization toolkit for STEPS simulations

The importance of visualization for spatial reaction-diffusion simulations is a matter of debate. Though visualization provides an intuitive way for understanding simple biochemical models, its value for simulations with complex biochemical systems and geometries is unclear. This leads to divergent strategies in existing simulators. Some simulators, for example Smoldyn and MesoRD, implement built-in runtime visualization support. Other simulators such as MCell focus on post-simulation result playback using third-party applications. Both approaches have their advantages and disadvantages, thus whether a simulator supports one over another mainly depends on developer preference and application focus. Runtime visualization provides immediate information of how the simulated system behaves, important for model debugging and runtime simulation adjustment. However, a considerable amount of computational resource is required, reducing the overall efficiency of the simulator. Moreover, modern neuroscience simulations are often executed on clusters where no visualization is allowed. Therefore, runtime visualization is often implemented as an optional feature that can be switched off when necessary. Post-simulation result playback does not affect runtime performance of the simulation significantly, although history data storage is required. The amount of history data increases proportionally to simulation time, making this approach resource-consuming for long simulations. In addition, result playback can only be visualized after a simulation is completed, so it is unable to perform runtime adjustment of the simulation.

STEPS implements a Python-based, interactive 3D visualization toolkit for spatial reaction diffusion simulations. Currently, the toolkit focuses on supporting runtime visualization, but simulation recording and playback will be added as extensions in the future. Despite the general understanding that visualization is limited to simulations with simple models and geometries and mostly for demonstration purposes, the STEPS visualization toolkit attempts to provide efficient, accurate and comprehensible visualization support for simulations with complex biochemical models and geometries, a goal that is not trivial to achieve. Here we detail the challenges encountered during the toolkit development and explain the solutions taken to tackle those challenges.

#### Component assembly strategy for visualization of complex biochemical models and geometry

The fundamental goal of the visualization toolkit is to visualize simulations with complex biochemical model and geometry. A major challenge lies in the presentation, that is, how to produce human comprehensible visual output of a complex system. Visualization support in existing simulators often adopt an “All-In-One” strategy, where all molecules as well as the full geometry are displayed in a single window. Although this approach may be adequate for models with several reactions and simple geometries, due to the limitation of human perception, the visual output of such a presentation soon becomes incomprehensible as the complexity of the system increases.

**Figure 2:**
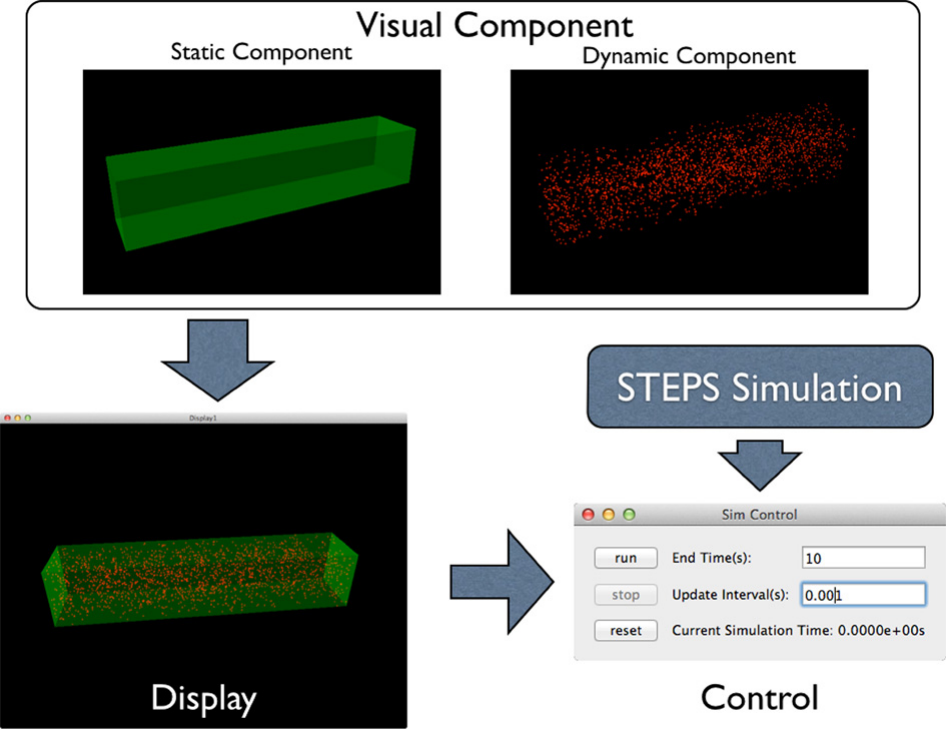
**Component Assembly strategy for the visualization toolkit**. Visual components, including static and dynamic component, are assembled in a display. The display is then assigned to a simulation control together with the visualized simulation.

To address this problem, the STEPS visualization toolkit abandons the “All-In-One” approach and introduces the “Component Assembly” concept to the implementation instead. Figure 2 provides an overview of the complete framework of the toolkit. The main building blocks in this implementation are “visual components,” which are independently functional Python classes for visualization of specified simulation data. Visual components can be divided into static components and dynamic components. Geometry of the simulation is represented by static components, including “compartment mesh” and “patch mesh,” which compartments and patches defined in the Tetmesh object can be associated with and visualized. These components are static since there is no further data update required once the components are created. Molecule changes that require constant updates during the simulation are represented by dynamic components. Several dynamic components are available for different visualization requirements. The “compartment species” component provides visualization of quantity and spatial changes of a given type of molecule species in a compartment, and the “patch species” component is the counterpart for molecule species on a patch. “Tetrahedron species” and “triangle species” components are the reduced version of the above two components, which display molecule changes within a list of tetrahedrons or triangles. In a STEPS simulation, species on patches are often composed to represent multiple-state channels, which switch between states depending on conditions such as membrane potential. These “channel species” can be visualized using the “patch channel” and “triangle channel” components. To distinguish between each other, each visual component has its own appearance configuration such as color and molecule size that can be either randomly generated or manually defined. Tetrahedral and triangular ROI datasets stored during geometry preparation can also be used to create respective species components.

Visual components are then assembled in a “display,” an interactive 3D window environment that displays assigned components. One pivotal feature of the visualization toolkit is the “Many-To-Many” association between visual components and displays: instead of creating a single display window, the toolkit allows multiple displays to be created for a single simulation. Multiple visual components can be assembled in a display and each visual component can also appear in multiple displays. Visual components that appear in multiple displays maintain a single instance of internal data and synchronize their visual appearance among all displays when the data is updated during simulation, thus the increase of memory cost is insignificant.

This implementation provides flexible solutions for different visual scenarios that may be encountered in practice. One common example is the “Global-ROI” scenario, where a single window displays all geometry components and molecule changes as a global view of system behavior, while a number of displays highlight changes of specific molecule species in different geometry regions. Another example is the “Species of Interest” scenario. In a complex simulation, molecules in different parts of the same geometry region often visually overlap with each other, significantly reducing the comprehensibility of the visualization. With the visualization toolkit, molecule species that are of interest can be isolated from the others and visualized in several displays separately, with the same static component as the geometry background of all displays.

Visual components and displays are extensions of generic OpenGL visual items provided by PyQtGraph (http://www.pyqtgraph.org/), a Python based scientific graphics and GUI library built on PyQt4 (http://www.riverbankcomputing.com/software/pyqt), PyOpenGL (http://pyopengl.sourceforge.net/) and NumPy. Visualization and interaction such as panning and rotation of views are handled directly by the package, allowing our implementation to focus on high level data representation instead of basic functionality coding. The package also supports runtime console interaction so that components can be added to or removed from displays to form new views of the simulation.

Displays with dynamic visual components need to be further assigned to a “simulation control” so that visualization can be synchronized with the simulation. Multiple simulation instances with different biochemical models and geometries can be assigned to a controller, where a background execution thread is generated for each of the simulations using Python's multithreading mechanism. Although all simulations are executed in parallel, they share a global configuration of simulation end time and visual update interval, which can be overwritten during simulation. The simulation control also unlocks the Global Interpreter Lock (GIL) in Python, thus users can interact with the visual system freely even when the simulations are in execution in the background.

#### Accurate representation of SSA-based spatial reaction diffusion simulations

Visualization of SSA-based spatial reaction diffusion simulation faces an intrinsic representation challenge that seldom appears in particle-based simulations, where the spatial position of each molecule is tracked and recorded accurately through simulation. The fact that SSA-based simulators do not track molecule movement but monitor the quantity changes of molecules in each subvolume means that the exact position of individual molecules is not known. Different approximations have been used to solve this problem. For instance, MesoRD allows users to predefine the maximum number of molecules that can be visualized per cubic subvolume. Based on this value, it then generates all possible molecule positions in advance by evenly partitioning the axes of the subvolume space. During simulation, each subvolume updates its condition iteratively and determines whether a molecule should appear on any of the positions. However, this approach was not suitable for STEPS visualization for several reasons. First, tetrahedral subvolumes have a much wider range of size and shape compared to the ones in a cubic mesh, thus it is practically difficult to partition the space evenly for each subvolume. Second, if all molecule positions are generated in advance it is possible for a molecule to be shown at a fixed position over time, giving the wrong impression that no movement has occurred for that molecule where instead conceptually it has changed position inside the subvolume. Third, as the maximum number of visible molecules is fixed for each subvolume, subvolumes with high concentrations of molecules may be visually over-simplified due to a lack of available positions, while the ones with low concentrations retain large amounts of unused coordinate data. Finally, the number of coordinates that need to be generated scales linearly with the number of subvolumes in the simulation, causing a large memory cost for simulations with fine meshes even if the amount of molecules in the system is small.

Because of these reasons, the STEPS visualization toolkit, instead, adopts a runtime generation approach for molecule visualization. At each visual update iteration, tetrahedral and triangular SSA subsystems in every dynamic visual component calculate the number of molecules within themselves and generate the exact number of corresponding random positions. The toolkit uses a fast algorithm that guarantees all these random positions are uniformly distributed and bound by the subsystem's geometry. These positions are then fed to individual visual components and rendered in the corresponding displays as dots with different sizes and colors, predefined in the component. The process repeats when the simulation reaches the next visual update interval. One exception is the multiple-state “channel species” on patches, whose positions are permanent after initialization except when they diffuse inside the membrane.

In the above solution, the number of random positions generated at each iteration equals the total number of molecules over all visual components. While this is achievable for simulations with a small numbers of molecules, as this number increases it becomes difficult and eventually unfeasible to render them due to limited graphical resources. Therefore the visualization toolkit regulates the position generation with two restrictions. The first restriction is the “maximum amount of points” that can be generated for each visual component. Once the number of molecules in a component exceeds this maximum, a reducing function is called to lessen the amount of points generated according to the second restriction: “maximum point density,” defined as the maximum number of possible points being generated per unit of measurement (m^3^ for tetrahedron, and m^2^ for triangle). For each associated tetrahedron/triangle of the visual components, the maximum number of points that can be generated within is determined by multiplying the density with its volume or area, reflecting the proportional distribution of molecules. The density can be either predefined by the user, or adjusted automatically according to the ratio of maximum against actual amount of points that will be generated when the auto-adjust mode in the reducing function is enabled. Each visual component has its own maximum amount and density configuration so that they can be specified for individual species but remain consistent within the component.

The runtime point generation approach requires fast data synchronization between visualization and simulation, which is often considered to be a weakness of pure Python applications. In our toolkit, this issue is managed by allowing direct data interfacing between STEPS and NumPy. NumPy is a Python extension package that supports large, multi-dimensional array construction and fast array operation. It is currently the standard Python package for numerical computing and is supported by many scientific computing packages. NumPy arrays are also the fundamental data structure for PyQtGraph, the package we used to implement our visualization toolkit. Using the SWIG interface, NumPy arrays can be directly accessed by other C/C++ packages, including STEPS. At each visual update cycle, molecule distribution data from the simulation is written directly in formatted NumPy arrays that will be assigned to visual components, eliminating expensive STEPS-Python-NumPy data copying. Our implementation also further speeds up the visual system by implementing all computational intensive routines, such as random point generation, in C++.

#### Quantitative visualization of simulations

Although the approach described above provides an intuitive grasp of how the simulation performs, information acquired from it is generally vague and qualitative. Important modeling information such as concentration and spatial distribution changes of molecules can only be observed with more accurate, quantitative analysis of the simulation. For this reason our visualization toolkit also implements a set of data plotting functions that enable dynamic monitoring of the amount and spatial distribution of molecules, which can be synchronized with the visualization updates during simulation. The quantitative plotting allows modelers to rapidly validate and debug their models at the early stage of model development, which is essential for complex computational models.

## Results

Application of the above toolkits highly depends on the conditions and research interests of specific projects. In this section we present two examples that originate from our previous research to explain how the toolkits can be used in practice. The meshes and Python scripts used for these simulations can be downloaded from ModelDB (http://senselab.med.yale.edu/modeldb/ShowModel.asp?model=153351). Video recordings of these two examples are provided as Supplementary Materials.

### IP_3_ receptor model

**Figure 3:**
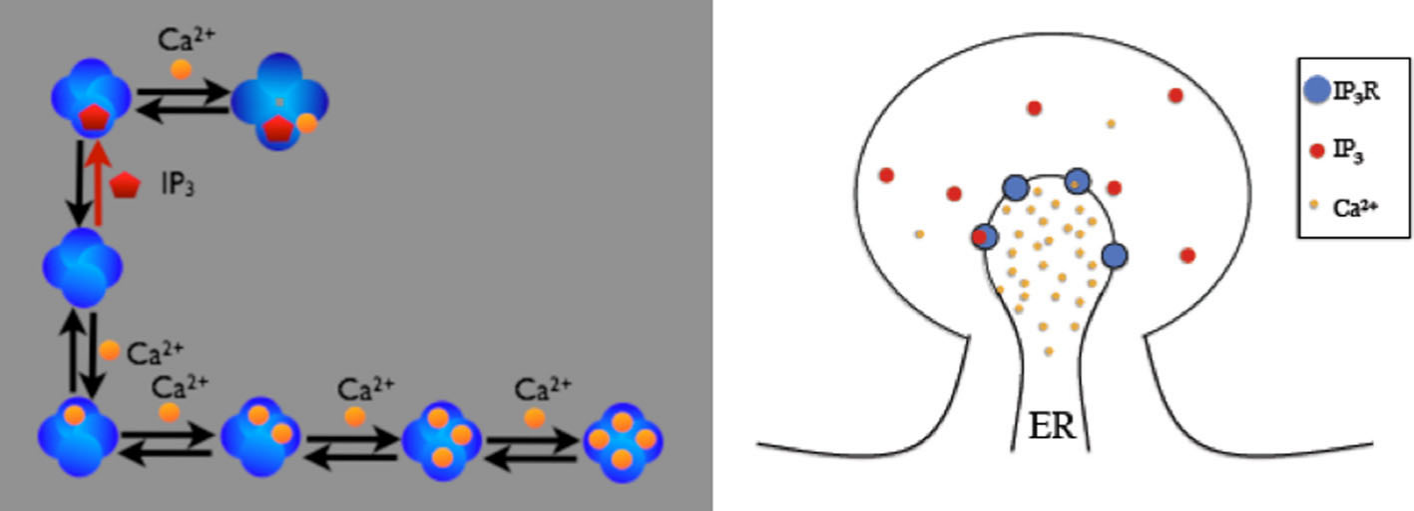
**Schematic description of the IP_3_R model on a spine**. IP_3_ receptors on the membrane can be opened by firstly binding with cytosolic IP_3_ then Ca^2+^, or can be inactivated by binding Ca^2+^ directly. Four inactivated states exist, depending on the number of Ca^2+^ ions bound to the receptor. Open IP_3_ receptors release Ca^2+^ from the ER into the cytosol.

The first example is the inositol 1,4,5-trisphosphate receptor (IP_3_R) model described in (Doi et al., 2005). In this model, IP_3_R on the membrane between Endoplasmic Reticulum (ER) and cytosol of a spine can be opened by first binding with cytosolic IP_3_ and then Ca^2+^, or can be inactivated by binding with Ca^2+^ directly. While open, IP_3_Rs release Ca^2+^ stored in the ER into the cytosol. Figure 3 provides a schematic illustration of the model. The goal of our example is to visualize the dependency between the existence of the open IP_3_R state and the Ca^2+^ concentration increase in the cytosol.

**Figure 4:**
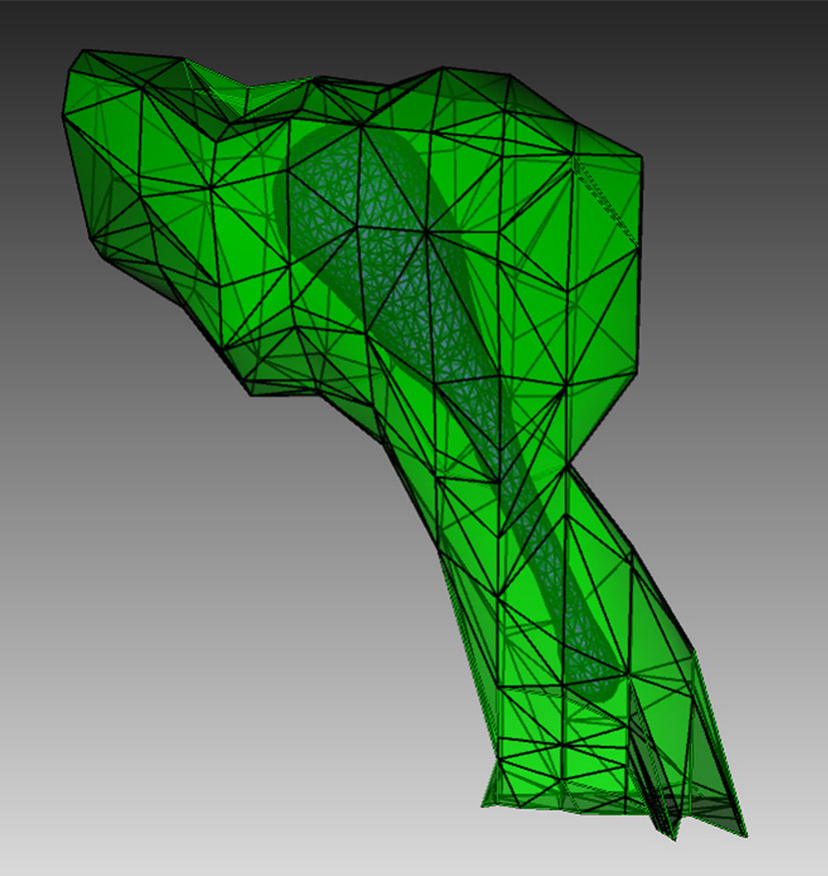
**Geometry for the IP_3_R model simulation**. Green: A spine morphology extracted from EM spiny dendrite reconstruction. Purple: triangular surface that divides the spine into ER and cytosol.

To create a suitable geometry for the simulation, we extract a triangular spine morphology from an electron microscopic reconstruction of spiny dendrites (http://synapses.clm.utexas.edu/anatomy/Ca1pyrmd/radiatum/K24/K24.stm) and artificially create a triangle mesh inside to represent the ER membrane of the spine (Figure 4). This combined triangular surface mesh is then converted to a tetrahedral mesh in CUBIT. The geometry preparation toolkit is used to create the Tetmesh object, which consists of two compartments: an inner compartment representing the ER and an outer compartment representing the cytosol, and a patch for the ER membrane. The compartments and patch are associated with the biochemical model where Ca^2+^ bindings and transitions of different IP_3_ receptor sites are represented as surface reactions on the patch, and Ca^2+^ as well as IP_3_ are set to be diffusible in the cytosol compartment and Ca^2+^ is also diffusible in the ER compartment.

In the visualization, Ca^2+^ in cytosol and ER is represented in orange, while IP_3_ in cytosol is represented in red, using the “compartment species” visual component. Different IP_3_ receptor sites on the membrane are represented as different states of a “patch channel” component with individual color and transparency configurations. Native and Ca^2+^ bound receptor states are colored in blue with different transparencies, while the IP_3_ bound state and the open state are colored in magenta with 20 and 100% opacity, respectively.

**Figure 5:**
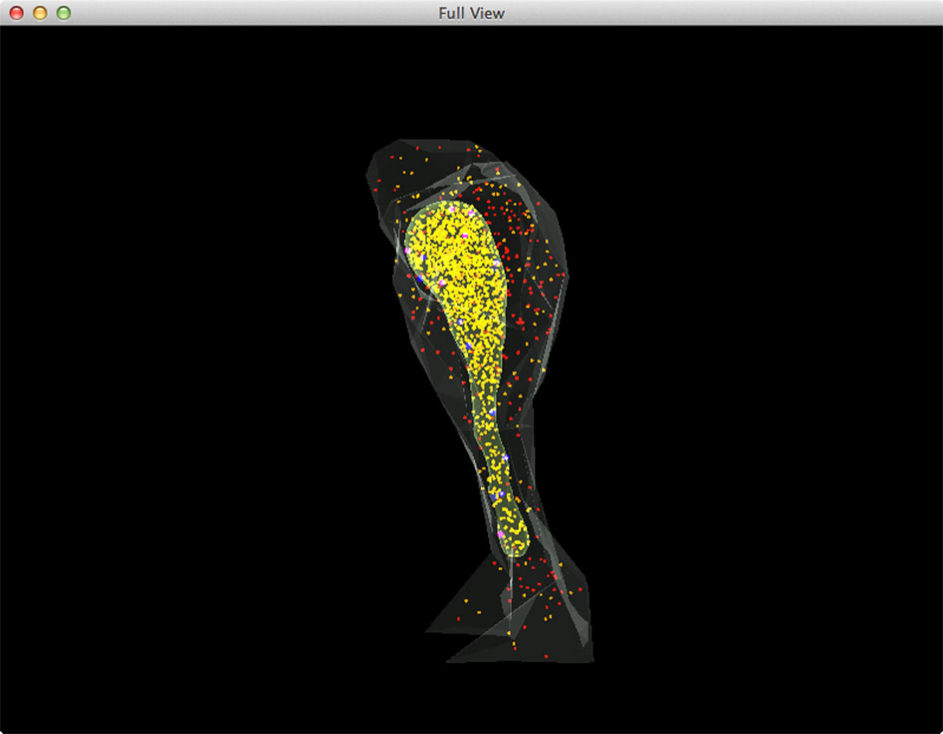
**A complete view of the IP_3_R simulation**. Red: IP_3_ in cytosol, Orange: Ca^2+^(Ca^2+^ in ER is rendered as yellow due to the color combination with ER compartment), Blue: inactivated IP_3_ receptors, Dark magenta: IP_3_ bound receptors, Bright magenta: IP_3_ receptors in open state.

**Figure 6:**
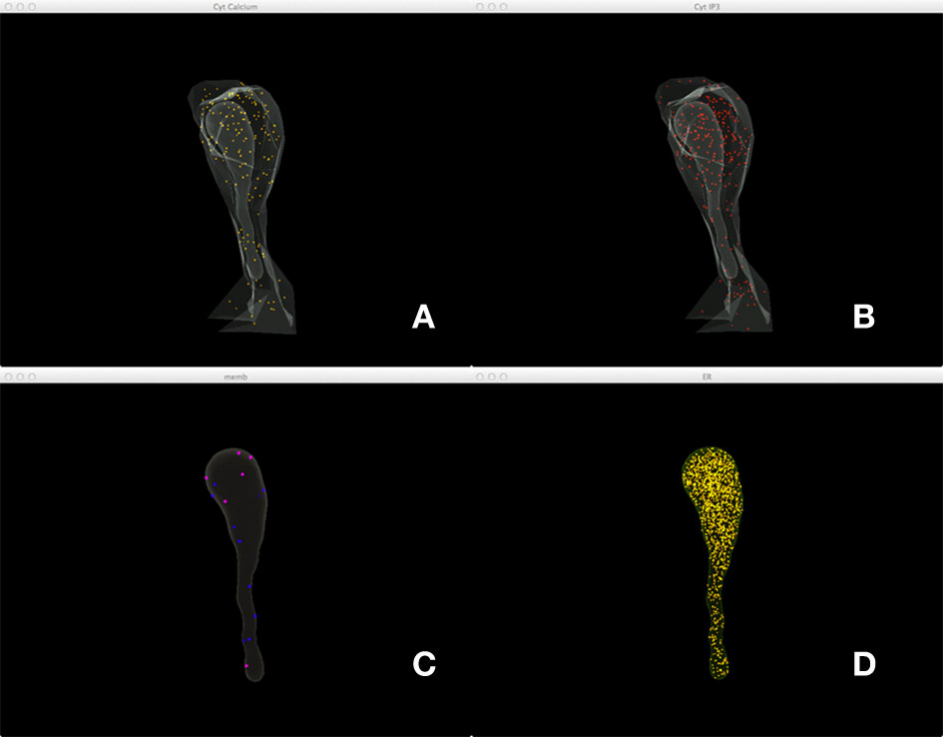
**A split view of the same simulation state as Figure 5. (A)** Ca^2+^ distribution in cytosol, **(B)** IP_3_ in cytosol, **(C)** IP_3_ receptors on the membrane with different states, **(D)** Ca^2+^ in ER.

Figure 5 shows a combined, “All-In-One” view of the simulation where all components are visualized in a single display, while an independent view of individual components at the same simulation state is provided in Figure 6 for comparison. Although multiple open receptors exist on the membrane, as confirmed in Figure 6C, they are invisible in Figure 5 due to the large number of Ca^2+^ ions and IP_3_ molecules present in the model. This is a common issue of visualization when dealing with complex simulations. Figure 6 provides an alternative solution where components are split and visualized in four different displays. In this solution, site transitions of IP_3_ receptor on the membrane can be clearly visualized in Figure 6C, while the increase of cytosolic Ca^2+^ concentration can also be seen in Figure 6A during simulation, thus the visualization is more comprehensible.

**Figure 7:**
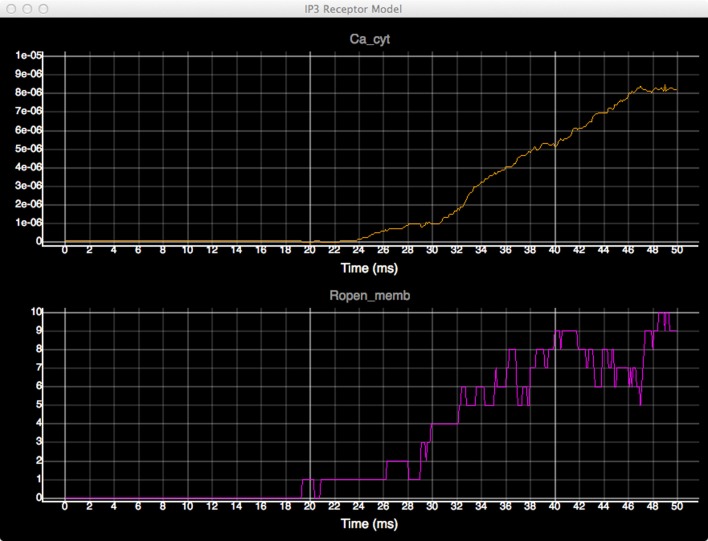
**Plots of cytosolic Ca^2+^ concentration (top) and the amount of IP_3_ receptors in open state (bottom) over time**.

In order to quantitatively analyze the relationship between the number of open states of the IP_3_ receptor and cytosolic Ca^2+^ concentration, we create dynamic plots with these two measures and monitor their changes throughout the simulation. As shown in Figure 7, the initial cytosolic Ca^2+^ activated an IP_3_ receptor at approximately 20 ms, leading to the release of Ca^2+^ from ER and the rapid increase of cytosolic Ca^2+^ concentration, which in turn increases the number of open-state IP_3_ receptors.

### Anomalous diffusion in spiny dendrites

The second example originates from our previous research (Santamaria et al., 2006, 2011) showing that molecules trapped by dendritic spines cause diffusion along spiny dendrites to be anomalous, with the degree of anomalousness proportional to spine density. This example aims to demonstrate this effect via diffusion simulations on simplified dendritic meshes with varying spine densities.

**Figure 8:**
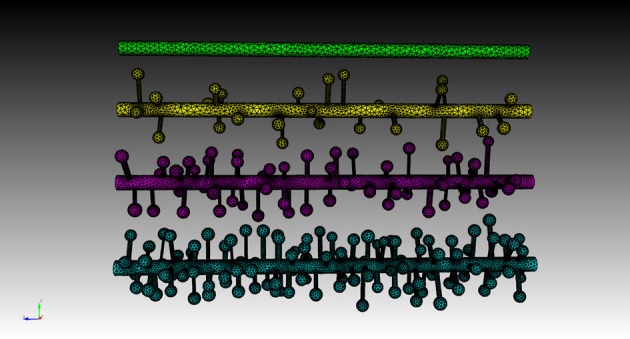
**Meshes of simplified dendrites with different spine densities**. Spine density for each mesh (in top–down order): 0, 2, 4, 8 spines/μm.

**Figure 9:**
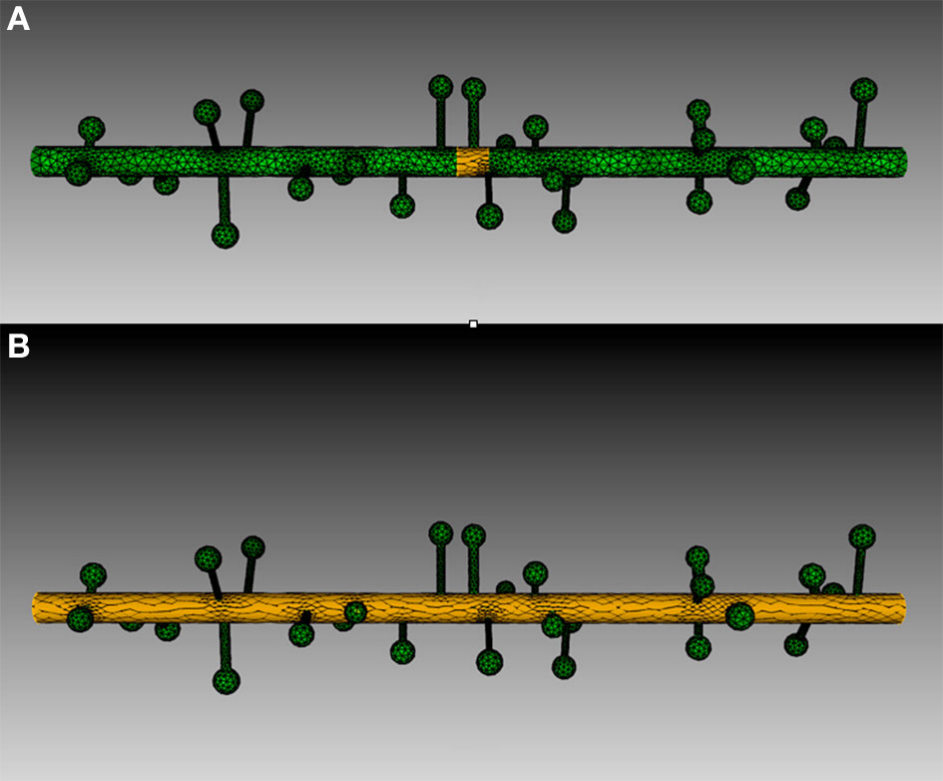
**Tetrahedron selection using geometry preparation toolkit of (A) Injection zone and (B) dendritic shaft**. A cylinder-bounding object is firstly created, as highlighted in the figure. Tetrahedrons in the mesh that are within the bounding object are selected by the toolkit and stored in the ROI dataset.

Four meshes were generated for this example, using project specified scripts for the CUBIT Python API. The mesh generation script is available upon request and can be modified to produce variations of the meshes. Each mesh consisted of a cylinder of 20 μm length and 0.7 μm diameter, representing the dendritic shaft. We then randomly attached a number of simplified spines, each formed by a spherical head and a cylindrical neck, onto the shaft cylinder. Spines were generated according to statistics from EM studies (Harris and Stevens, 1989) and distributed randomly along the shaft cylinder with densities varying from 0 (as smooth dendrite) to 8 spines/μm length. Figure 8 gives an overview of these meshes. A biochemical model with one diffusible molecule species is associated with the meshes. We initialize each simulation by injecting 2000 molecules into a cylindrical zone of 0.7 μm length and 0.7 μm diameter at the center of each shaft cylinder. This can be achieved using the indirect element selection method described previously (Figure 9A). Tetrahedrons chosen by the selector are stored in the ROI dataset of the corresponding Tetmesh object. Using a similar approach we also select and store indices to all tetrahedrons within the shaft cylinder (Figure 9B) and use them in later visualization.

**Figure 10:**
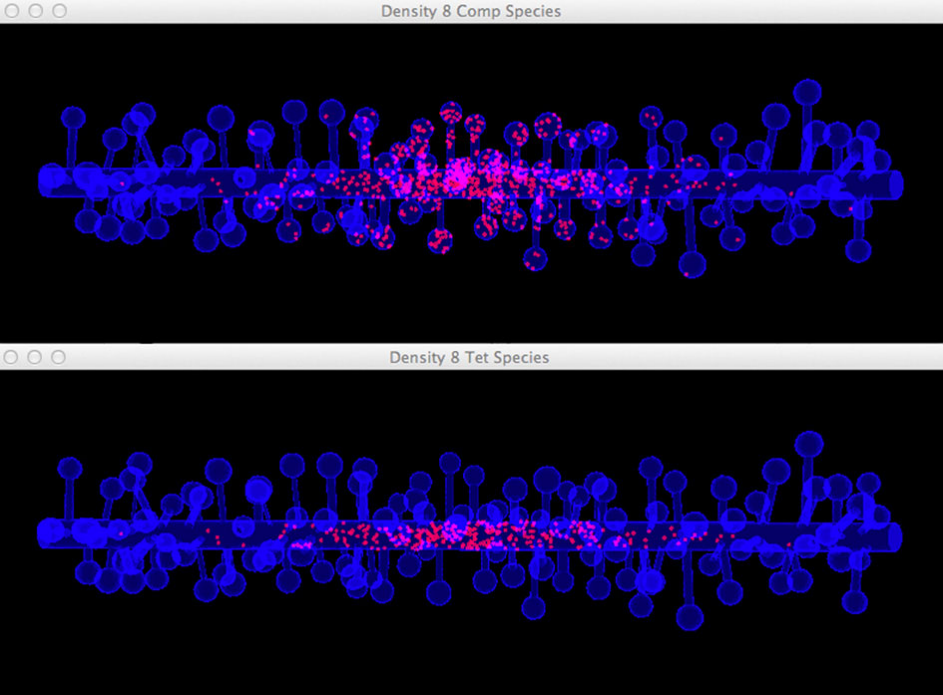
**Comparison between the visualization of Top: compartment species component and Bottom: tetrahedron species component in anomalous diffusion simulation with the 8/μm spine density mesh**. Molecules that are trapped in spines are displayed in the compartment species component, but are filtered by the tetrahedron species component. The latter produces a more clear view of molecule distribution in the shaft cylinder.

**Figure 11:**
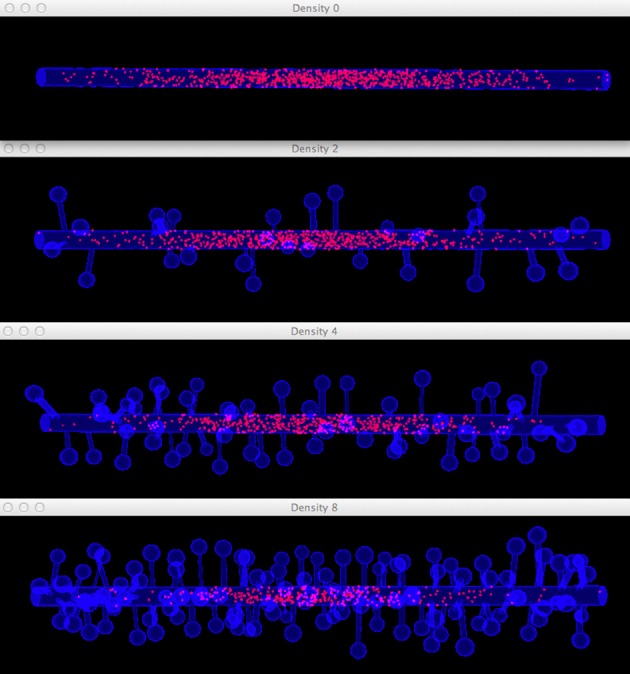
**Comparison of diffusion simulations in meshes with different spine densities**. As the spine density increases molecules can clearly be seen to diffuse more slowly along the dendritic shaft, indicating the anomalous diffusion effect.

Four simulations are assigned to and executed by a simulation control, each of which simulates molecule diffusion in one of the four meshes. States of the simulations are visualized in separated displays. In each display, the mesh for the simulation is rendered by the compartment mesh component. As this research mainly focuses on the molecule distribution in the dendritic shaft, we use the shaft tetrahedron indices stored in the ROI dataset to create a tetrahedron species component that only displays molecules inside these tetrahedrons. This is a better solution compared to the one where all molecules in the simulation are displayed, particularly for meshes with high spine densities (Figure 10). Visual updates of displays are synchronized by the simulation control so that their results are visually comparable. As shown in Figure 11, noticeable difference of molecule distribution in dendritic shafts can be observed after a period of simulation, indicating the anomalous diffusion effect.

**Figure 12:**
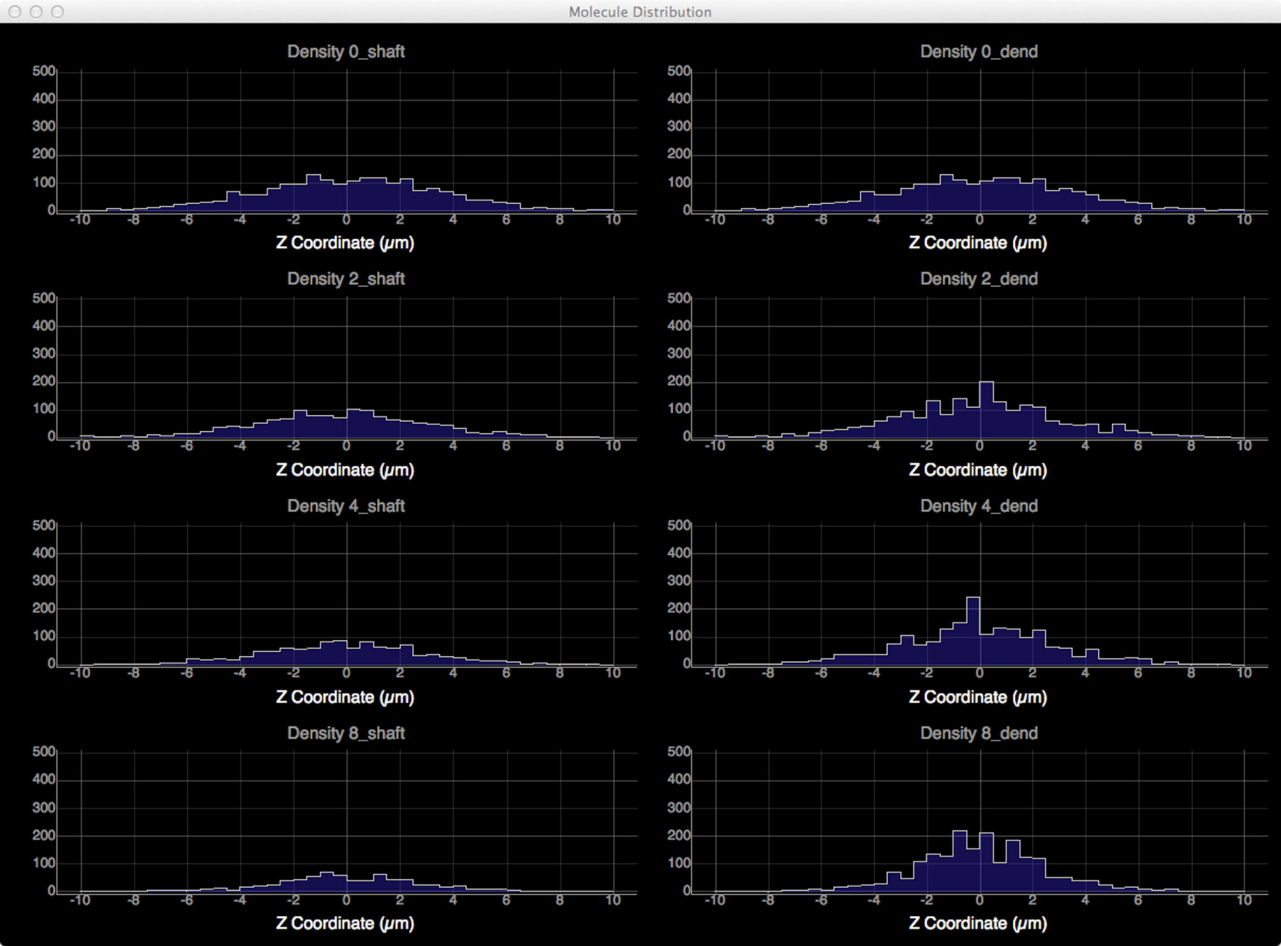
**Spatial distributions of molecules along dendritic shafts for simulations with varying spine densities (in top–down order: 0, 2, 4, 8 spines/μm)**. Left: distributions of molecules in the dendritic shafts. Right: distributions of molecules in the whole dendrite. As spine density increases, more molecules are trapped in spines, significantly delaying the diffusion along dendritic shafts.

To quantitatively visualize the difference of molecule distribution caused by varying spine density, we plot the spatial distributions along dendritic shafts using the visualization toolkit (Figure 12). With increase of spine density, more molecules become trapped in the spines and are thus are unable to diffuse along the dendritic shaft. The distribution result corroborates our previous study (Santamaria et al., 2006, 2011).

## Discussion

In this paper we have described two supporting toolkits for STEPS that are implemented in Python. We've introduced the geometry preparation toolkit that integrates CUBIT with STEPS via Python, allowing complete mesh preparation solutions for STEPS simulations. We've also analyzed approaches to improve efficiency, accuracy and comprehensibility of visualization for spatial reaction diffusion simulations, which are adopted in our implementation of the visualization toolkit. Two examples are presented to showcase the application of the toolkits in real research projects. The IP_3_R model example demonstrates how compartments and patches are identified and created in realistic spine morphology using the geometry preparation toolkit, and how the simulation can be visualized properly by splitting molecule species in multiple displays. The anomalous diffusion example showcases the usage of “Regions of Interest” datasets for visually filtering molecules in a specific region. This example also demonstrates how multiple simulations are executed and visualized simultaneously for comparison.

The toolkits are components of the STEPS supporting environment, where Python-based submodules are implemented to close the gaps between interfaces of various Python packages and the generic interface of STEPS. The Python world is an open and rapidly growing community where hundreds of new packages are available to the public everyday. On one hand, this provides rich and flexible package options for research projects using STEPS, on the other hand, packages selected to implement a customized toolkit may soon be out of date or lack improved features provided in new packages. Therefore, instead of detailing the package-dependent, technical implementation of the toolkits, we've concentrated on introducing novel, underlying mechanisms and principles involved. The approaches described in this paper are beneficial not only to the implementation of current toolkits, but also to the design and implementation of toolkits for other simulators in the same category.

At the moment the STEPS supporting environment is not yet completed, and the existing toolkits can be further improved in several aspects. The generation of biochemical models remains text based, requiring significant amount of human efforts in scripting and maintenance, despite the availability of the SBML (Hucka et al., 2003) import introduced since STEPS ver. 1.2 (Hepburn et al., 2012). A graphical model description and generation system would therefore be beneficial. Data gathering and recording have not yet been included in STEPS, thus result analysis still greatly relies on inefficient, non-generic Python scripting by the individual user. A data recording system is in development, where data in STEPS simulations can be stored directly in formatted NumPy arrays via the SWIG interface described before, according to user-defined recording schedules.

As for the toolkits described in this paper, the geometry support toolkit requires CUBIT, which is commercially licensed. We anticipate alternatives with similar functionality that can be obtained freely so that the whole geometry preparation process can be achieved without extra financial cost. One candidate is TetGen, whose format has been supported in STEPS since early versions, although it still lacks several features such as graphical interaction with meshes. So far, the visualization toolkit supports visualization of spatial reaction diffusion systems, but does not yet support visualization of new features in STEPS version 2, such as membrane potential and current, which is implemented in the EField system (Hepburn et al., 2013). This can be achieved by implementing new visual components within the current toolkit framework. We are also investigating how to further speed up the real time 3D rendering, which is essential in the support of large-scale simulation visualization.

STEPS 2.2 with both toolkits described in this paper, as well as API references and a user manual, can be accessed from http://steps.sourceforge.net.

## Author contributions

Weiliang Chen designed, implemented and tested the toolkits described, as well as drafted the manuscript. Erik De Schutter conceived of and supervised the STEPS project and helped draft the manuscript. Both authors contributed to the manuscript and read and approved the submission.

## Conflict of interest statement

The authors declare that the research was conducted in the absence of any commercial or financial relationships that could be construed as a potential conflict of interest.

## Supplementary material

Movie 1 **Simulation recording of the IP3 Receptor example**.

Movie 2 **Simulation recording of the Anomalous Diffusion example**.
